# The Bristol Hospital for Sick Children

**Published:** 1898-04-09

**Authors:** 


### THE BRISTOL HOSPITAL FOR SICK CHILDREN.

The charges brought by Dr. Christie, formerly house surgeon at the Bristol Hospital for Sick Children, against the medical staff, the management, and the nursing of the hospital, having been fully investigated by a Commission appointed by Lord Herschell, have been declared to be untrue and not justified. According to the statement made by the President of the Commission of Inquiry, the charges made by Dr. Christie were very grave, and no one taking up the pamphlets which he had circulated among the subscribers and the public could fail to come to the conclusion that they were as grave as any could be against the staff of a hospital. Any ordinary person reading it would come to the conclusion that the cases mentioned gave evidence of gross cruelty, ignorance, and incompetence on the part of the staff. Dr. Christie had suggested that he was fighting the battle of the new surgery against the old, but the Commissioners had come to the conclusion that in publishing these pamphlets he was not fighting anything of the kind, and that there was no reason in this hospital that he should. In regard to the fact that Dr. Christie had chosen to retire from the inquiry, they pointed out the law on the subject, which was to the effect that, their arbitration having been accepted by both parties, a submission such as that, unless a contrary intention was expressed therein, was irrevocable except by leave of the High Court or a judge, and would have the same effect as if it had been made an order of the Court. The award of the arbitrators was therefore final and binding upon the parties. The award was to the following effect: (1) (As to the charge that the medical and surgical treatment of children by the staff was conducted with cruelty, neglect, ignorance, and incompetence, as evidenced by certain flagrant cases, some of which, in consequence, resulted in death). This charge was untrue, and not justified as fair and proper criticism upon any acts done or performed by any of the persons charged. (2) (That the nursing of the children in the hospital was characterised by cruelty, neglect, and incompetence.) Of this charge, part was declared to have been withdrawn and admitted to be a mistake; the other part was untrue and not justified. (3) (That the management of the hospital was badly conducted, and that the regulations were defective.) As to this, there was no evidence of bad or improper management, except in so far that the hospital authorities did not dismiss or effect the resignation of Dr. Christie at an earlier date. (4 and 5) (As to dishonesty on the part of the President and Committee of the hospital by hoodwinking the public, and as to falsifying or tampering with the notes.) That these charges were untrue and not justified.

